# Automated cleaning and pre-processing of immunoglobulin gene sequences from high-throughput sequencing

**DOI:** 10.3389/fimmu.2012.00386

**Published:** 2012-12-28

**Authors:** Miri Michaeli, Hila Noga, Hilla Tabibian-Keissar, Iris Barshack, Ramit Mehr

**Affiliations:** ^1^The Mina and Everard Goodman Faculty of Life Sciences, Bar-Ilan UniversityRamat Gan, Israel; ^2^Department of Pathology, Sheba Medical Center and Sackler School of Medicine, Tel Aviv UniversityTel Aviv, Israel

**Keywords:** B cell receptor, computer programs, high-throughput sequencing, immunoglobulin (Ig) genes, insertions and deletions (indels)

## Abstract

High-throughput sequencing (HTS) yields tens of thousands to millions of sequences that require a large amount of pre-processing work to clean various artifacts. Such cleaning cannot be performed manually. Existing programs are not suitable for immunoglobulin (Ig) genes, which are variable and often highly mutated. This paper describes Ig High-Throughput Sequencing Cleaner (Ig-HTS-Cleaner), a program containing a simple cleaning procedure that successfully deals with pre-processing of Ig sequences derived from HTS, and Ig Insertion—Deletion Identifier (Ig-Indel-Identifier), a program for identifying legitimate and artifact insertions and/or deletions (indels). Our programs were designed for analyzing Ig gene sequences obtained by 454 sequencing, but they are applicable to all types of sequences and sequencing platforms. Ig-HTS-Cleaner and Ig-Indel-Identifier have been implemented in Java and saved as executable JAR files, supported on Linux and MS Windows. No special requirements are needed in order to run the programs, except for correctly constructing the input files as explained in the text. The programs' performance has been tested and validated on real and simulated data sets.

## Introduction

Studying the generation, development and selection of lymphocyte repertoires, and their functions during immune responses, is essential for understanding the function of the immune system in healthy individuals, and in monitoring and intervening with the immune system in immune deficient, autoimmune disease or cancer patients. The recent development of high-throughput sequencing (HTS) enables researchers to obtain large numbers of sequences from several samples simultaneously (Galan et al., 2010). HTS has a great advantage over classical sequencing methods in the field of immunoglobulin (Ig) gene research, as it enables us to extract more sequences per sample and it is sensitive enough so we can identify different unique sequences. Ig genes encode the B cell receptors (BCR) and tend to accumulate point mutations in their sequences in order to improve the BCRs affinity to antigens. Mutation analysis, which is one aspect of Ig gene research, enables the tracking of mutation accumulation in the BCRs and hence analysis of the diversification of Ig gene sequences that originate from the same ancestor. Thus, HTS presents us now, for the first time, with the ability to analyze and compare large samples of mutated Ig gene repertoires in health, aging and disease (Campbell et al., 2008; Boyd et al., 2009; Gibson et al., 2009; Scheid et al., 2009; Ademokun et al., 2010; Dunn-Walters and Ademokun, 2010). However, the huge numbers of sequences obtained require a large amount of pre-processing work to clean out artifacts, sort sequences according to sample according to their molecular identification (MID) tags, identify primers, and discard sequences that do not contain enough information, such as sequences much shorter or longer than the expected length of an Ig variable region gene, or sequences with average quality scores below a defined threshold. Several programs are already used by the scientific community to study the B cell mutational patterns, such as SoDA (Volpe et al., 2006), SoDA2 (Munshaw and Kepler, 2010), and iHMMune-Align (Gaëta et al., 2007) which perform V(D)J segment identification; identification of clonally-related Ig gene sequences using clustering methods (Chen et al., 2010); ClustalW2 (Larkin et al., 2007), for alignment of clonally-related sequences; and IgTree (Barak et al., 2008), for creating lineage trees from the sets of aligned clonally-related sequences. However, in order to use these programs and receive reliable results that are not affected by sequencing artifacts, one must first make sure that all such artifacts are cleaned out of the input data.

Although HTS has already been available for several years, there are very few such cleaning programs available for users, and none that can deal with the cleaning of Ig genes. For example, the program CANGS (Pandey et al., 2010) has a very good pipeline of cleaning sequences, but it discards unique sequences and searches for primers and MID tags with perfect matches only, while it is known that tags and primers are often incomplete or sequenced incorrectly. Another program that could be used for Ig gene data cleaning is SeqTrim (Falgueras et al., 2010). This program does all the desired cleaning processes, but has two main disadvantages: one is that it runs on sequences that were inserted in vectors, so the program identifies the inserts only according to vector sequences found in databases such as NCBI's UniVec, EMBL's emvec, or BLAST. The user does not have the option to insert the ends of the genes, e.g., primers, as an input. Therefore, this program is suitable only for researchers that use sequencing with vectors. A second disadvantage is that SeqTrim requires other external programs. Ngs_backbone (Blanca et al., 2011) is another program that can be used for cleaning Ig gene sequences, but it requires external softwares. Additionally, there are several programs that trim the adapters (primers used in HTS) and the template-specific primers (MID tags or barcodes). One of them is TagCleaner (Schmieder et al., 2010), but this program takes all the input sequences, aligns them in order to identify the most frequent sequences at both ends that are supposed to be the adapters/tags and trims them. TagCleaner and similar programs are therefore ineffective when sequences come from several samples and hence contain several tag combinations; tags are composed of different sequences, so no consensus sequence can be deduced correctly using this method. In addition, highly homologous or, in contrast, highly variable sequences may yield erroneous alignments and therefore false identification of adapters. We have tested TagCleaner on our Ig genes data but this program could not correctly identify the tags. Another program is TagDust (Lassmann et al., 2009), but this program identifies artifact reads by comparing all reads to a library of sequences and checking for significant match. It is not possible to create such library for Ig gene sequences, as they undergo somatic hypermutation (SHM) in a high rate, and thus can diverge (Cook and Tomlinson, 1995; Rajewsky, 1996). EA-utils (Aronesty, 2011), Scythe (Buffalo, n.d.), SeqPrep (John, n.d.), FASTX (Gordon, n.d.), and Trim Galore! (Krueger, n.d.) are additional programs that are used to trim adapters among other functions; however, it is not possible to identify adapters in the 5′ end of the reads and to search for multiple different adapters using these programs. Using trimLRPatterns [one tool of ShortRead, (Morgan et al., 2009)] lacks the option to search for multiple different adapters. Trimmomatic (Bolger and Giorgi, n.d.) does not allow identifying adapters in the 5′ end of the reads, and anyway is compatible to Illumina sequencing only. FAR [The Flexible Adapter Remover, (Unknown, n.d.)] is capable of searching for multiple adapters using a simple global alignment algorithm, but it does not record the combination of adapters (or barcodes or MIDs) if found and cut. This is important when sequencing several different samples in the same sequencing run. Cutadapt (Martin, 2011) offers an easy-to-use command-line program that searches for multiple adapters and trim them, and is specialized for small RNA sequences. However, cutadapt currently does not support using a configuration file in which, for example, a list of adapters can be specified; hence, inputting several adapter sequences is via the command-line, which makes it slightly cumbersome. AdapterRemoval (Lindgreen, 2012) can search for multiple adapters in both 5′ and 3′ ends of the reads and discards reads that do not exceed a minimum length given by the user. However, none of the above mentioned programs assign the reads to their original samples according to their MIDs (barcodes), although the search of adapters should be similar to the identification of MIDs. Btrim (Kong, 2011) presents the closest cleaning options to our desired ones. In addition to trimming adapters and low quality regions as some of the above programs do, it can also identify barcodes and assign the reads to their original samples. However, Btrim has several shortcomings. First, it is limited to Linux. Second, similar to some of the above-mentioned programs, it requires some knowledge regarding the use with the command-line. A program with a user interface or even a double-click program is preferable, as its use can be included in an automated pipeline easy to operate even by users with little experience with computers. Moreover, it can search for multiple adapters or barcodes, and it can work with a configuration file containing all the adapters or barcodes to search, but it requires this file to contain pairs of 5′ and 3′ adapters or barcodes. This way, if barcodes were used in several combinations for samples, as we do, this file should contain all possible combinations of barcodes.

Thus, we needed—and created—a program that can clean the sequences of artifacts, and would be suitable for use with Ig genes in spite of their unique characteristics. We present here the Ig-HTS-Cleaner program, which enables the user to give the ends of the genes (primers and MID tags) as input no matter what their origin is, can handle multiple tags, and does not require any additional programs in order to run. Our Ig-HTS-Cleaner program does not require any knowledge in programming nor complicated installation, only a simple input file which contains the parameters for run. Moreover, the FASTA output files enable easy downstream analyses of the sequences.

Sequencing of complete Ig genes can currently be carried out only by the 454 pyrosequencing sequencing platform or the illumina platform. The reason is the maximum sequencing length required in order to get the full Ig gene. Only 454 or illumina currently reach a maximum read length of 500 nucleotides. Other platforms can reach such lengths only by using the paired-ends method, when only the ends of the gene are sequenced and the middle is inferred by comparing to a reference gene. This, of course, is not valid with Ig genes, due to their huge variability and the lack of a reference gene. Other platforms require assembly of complete sequences from shorter reads, which is also a problem due to the high mutation load and large numbers of similar but not identical sequences in Ig genes. When other sequencing platforms reach the same read length, our programs may be used on the data generated by them as well.

One of the shortcomings of pyrosequencing is that during the sequencing of homopolymer tracts (HPTs, repeats of the same nucleotide), the polymerase can add or delete one or more nucleotides from these repeats, or alternatively, the signal of poly-nucleotide incorporation can be misread (Huse et al., 2007). These errors may result in insertions/deletions (indels) that are a result of the sequencing and therefore are considered as artifacts (Margulies et al., 2005). In Ig gene research, it is very important to distinguish between artifact indels and legitimate indels that are a result of normal SHM and affinity maturation of B cells, although naturally occurring indels are very rare. Legitimate indels should be taken into account when analyzing mutations of the B cell Ig genes, and artifact indels should be discarded from the analysis. There are several common approaches for dealing with indels. Campbell et al. used an algorithm that discards sequences with insertions, deletions or substitutions that occurred near or in HPTs, unless the indel or the substitution was seen in both the forward and reverse reads (Campbell et al., 2008). In other studies, all sequences with any type of indel are excluded from analysis (Boyd et al., 2010; Wu et al., 2010) or included without accounting for indels (Wu et al., 2010). CANGS (Pandey et al., 2010) identifies indels that appear only in HPTs near the primers and discards them. The program does not identify indels occurring far from the primers. VarScan (Koboldt et al., 2009) and VARiD (Dalca et al., 2010) can identify indels, but these programs deal with variability and single nucleotide polymorphism (SNP) identification, and do not distinguish between legitimate and artifact indels, and are hence less suitable for identifying and discarding artifact indels from Ig genes. Recently, two methods for distinguishing true indels from sequencing artifacts have been developed. Dindel (Albers et al., 2011) utilizes a probabilistic method that accounts for the increased indel rates near HPTs. However, Dindel detects indels from short reads generated by Illumina sequencing and aligns the reads to a specific region in the genome. Hence, Dindel is not suitable for Ig genes because they are longer than Illumina reads and they cannot be aligned to a specific region in the genome due to the enormous variability and randomness of Ig gene rearrangements. PiCALL (Bansal and Libiger, 2011) also detects indels using a probabilistic method, but it works on a population of diploid individuals. Therefore, piCALL is not suitable for Ig gene sequences, in which different cells have different Ig genes.

Sometimes, discarding all the indels causes loss of information. For example, if an indel appears in a unique sequence, most of the algorithms would discard this sequence because no other sequence contains this indel. Nevertheless, there is a chance that this indel is legitimate, and we refer to such cases as uncertain indels. Because in several of our studies we perform sequencing on DNA from preserved tissue samples which do not yield large enough amounts of DNA (Tabibian-Keissar et al., 2008), we do not want to simply discard all unique sequences. Since there is no absolute way to identify all sequencing errors, and because we have limited sequencing data, we intend to save as many sequences as possible, and still avoid as many artificial indels and point mutations as possible. To address this issue, we developed Ig-Indel-Identifier, a program that identifies legitimate and artifact indels and does not discard sequences that contain uncertain indels. Hence, we may decide to keep these uncertain sequences for maximal information utilization.

This paper describes both Ig-HTS-Cleaner and Ig-Indel-Identifier. Our programs were designed to process Ig genes, but they are easily applicable to all types of sequences.

## Materials and methods

Ig-HTS-Cleaner and Ig-Indel-Identifier have been programmed in Java on the Windows operating system and is saved as an executable JAR file. The JAR files support Linux and Microsoft Windows. An executable file for each of our programs is available at http://immsilico2.lnx.biu.ac.il/Software.html. To execute the program, the user should double-click on the program symbol after saving it in the same directory where the input files are saved. No special requirements are needed in order to run them, except for correctly constructing the input files as explained below.

### Ig-HTS-cleaner input and output

**Figure 1:**
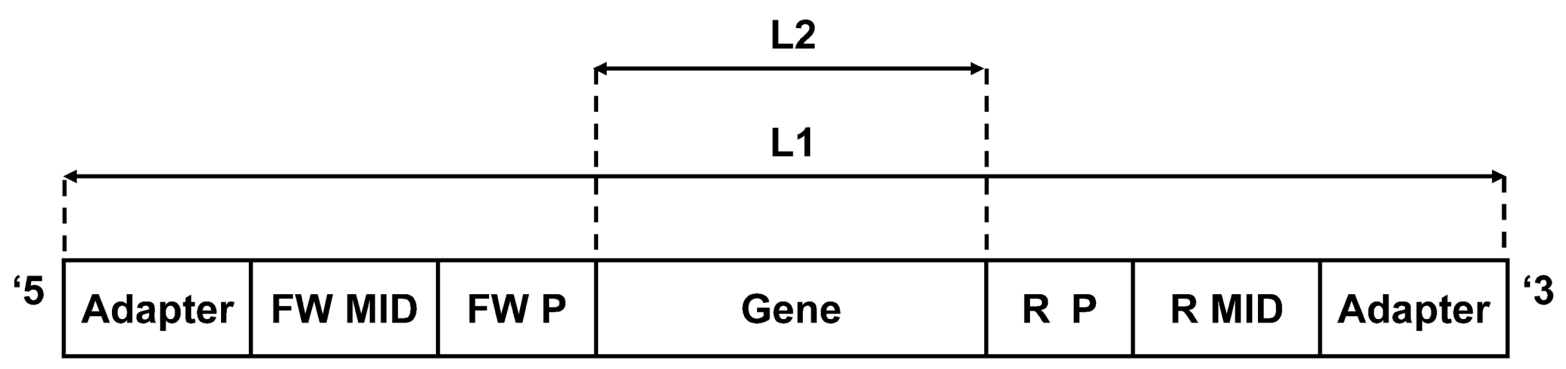
**A typical sequencer output (e.g., 454) read consists of the following segments (ordered from the 5′ end to the 3′ end):** adapter: sequencer adapters. FW MID denotes the forward molecular identifier (tag). FW P denotes the forward polymerase chain reaction (PCR) primer. Gene is the target sequence. R P denotes the reverse PCR primer. R MID denotes the reverse molecular identifier (tag). L1: Sequence length before first filtering—should be in the allowed range defined by the first minimum and maximum values (minL1 < L1 < maxL1, for example, 200 and 400 in Figure 2). L2: Sequence length after primers were cut—should be in the allowed range defined by the second minimum and maximum values (minL2 < L2 < maxL2, for example, 150 and 360 in Figure 2). These ranges can be changed in the input file according to the data set and the specific requirements of each study.

**Figure 2:**
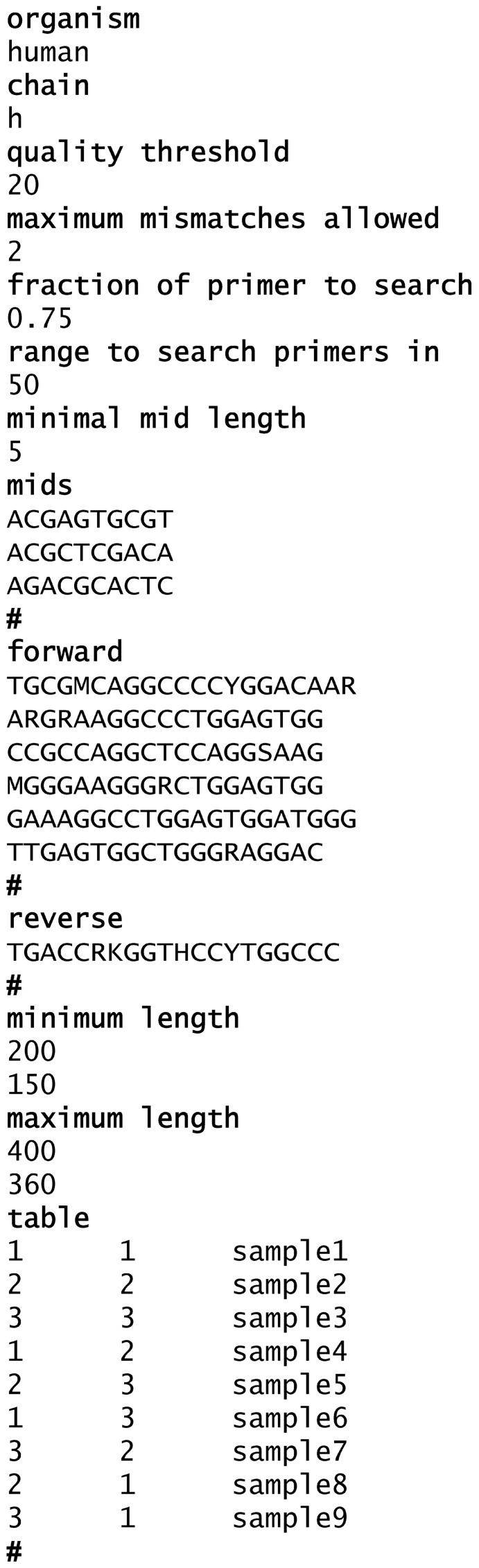
**An example of the input.txt file content.** In bold- words/characters that should always appear. **Organism**—represents the organism to which the sequences belong, “**Human**” in this example. **Chain**—represents the chain of the Ig (h, heavy as in this example; l, lambda; k, kappa). **Quality threshold**—the minimal average score for a sequence allowed. **Maximum mismatches allowed**—the number of mismatches the user allows when primers are being searched. “2” means that when primers are being searched, the sequence can contain 2 insertions/deletions or substitutions in the primer's sequence. **Fraction of primer to search**—in case the full primer was not found, the program searches only the given fraction of the primer from the side closer to the gene. **Range to search primers in**—the search is executed on a limited range of bases at the ends of the read. **Minimal MID length**—in case the full MID was not found, the program searches for a perfect match of the minimal length of the MID from the side closer to the gene. **Mids**—a list of the MID tags that have been used in the current sequencing. The program automatically numbers the MID tags according to the insertion order. At the end of each list, a “**#**” should appear, see example. In case no MIDs were used, leave only the title and the “#”. **Forward**—a list of the forward primers that have been used in the current sequencing, used for identification of the primers. At the end of each list, a “#” should appear, see example. **Reverse**—a list of the reverse primers that have been used in the current sequencing, used for identification of the primers. At the end of each list, a “#” should appear, see example. **Minimum length**—two values, the first is the minimal length for the first filtering of the data (minL1). The second value represents the minimal length that is legitimate for the genes in between the primers (minL2). **Maximum length**—two values, the first is the maximal length for the first filtering of the data (maxL1). The second value represents the maximal length that is allowed for the genes in between the primers (maxL2). **Table**—contains the MID tag combination per each sample that was sequenced. MID tag numbers should coordinate with their serial number in the above list. This enables the program to attribute each sequence to its corresponding sample. Each line should contain: number of the forward MID tag/tab/number of the reverse MID tag/tab/sample id (see example). At the end of each list, a “#” should appear, see example. In case no MIDs were used, put 0 as the number of forward and reverse MIDs.

The program receives as an input the following files, which should be saved in the same directory as the program. (1) A group of ^*^.fna files (^*^denotes any desirable name) containing the FASTA sequence reads—see Figure 1 for the structure of a typical read. (2) Quality (^*^.qual) files containing the scores for the sequences (both file types are received from the sequencing platform). (3) An input.txt file created and updated by the user, containing the parameters for the cleaning, such as the MID tags and the primer sequences, the length range within which the sequence is considered legitimate, the samples (in case more than one sample was sequenced), etc. A detailed explanation on how to fill the input.txt file can be found in Figure 2. The input file should be created precisely according to these instructions.

**Figure 3:**
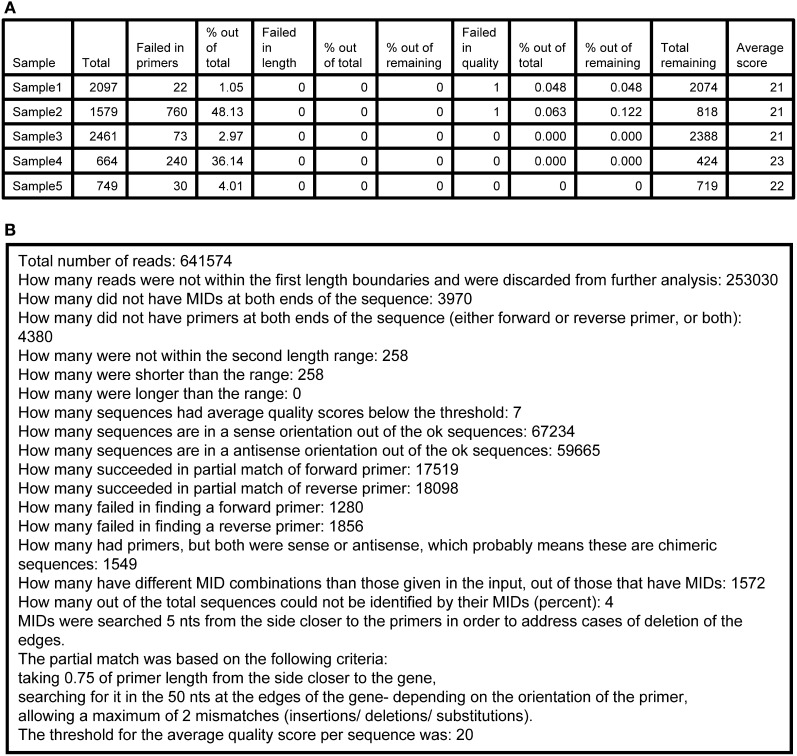
**A sample of an Ig-HTS-Cleaner log.txt output file. (A)** The first part of the file (after importing the .txt file into a table). “Sample” is the sample name, inserted in the input file. “Total” represents the total number of sequences received from the sequencer before cleaning (as counted after MID identification). “Failed in primers” represents the number of sequences without primers at both ends. “% out of total” means the % of the “Failed in primers” column out of the total column. “Failed in length” represents the number of sequences with length not in between the input range. “% out of total” means the % of the “Failed in length”column out of the total column. “% of the remaining” for the “Failed in length” column represents the % after we subtract the number of sequences failed in primers from the total and calculate the % from that. “Failed in quality” represents the number of sequences with an average quality score below the threshold. “% out of total” means the % of the “Failed in quality” column out of the total column. “% of the remaining” for the “Failed in quality” column represents the % after we subtract the number of sequences failed in primers and in length from the total and calculate the % from that. “Total remaining” represents the number of sequences that have passed all cleaning steps successfully. “Average score” is the average score for the sample. First, the average score for each sequence is calculated by an average of the scores per base, given in the .qual files in the 454 output. Then, the average score of the sample is calculated as the average of the scores of all sequences belonging to the sample. **(B)** The second part of the file, containing information and statistics regarding the run.

As output, the program generates the following files:

1. FailedInFindMIDs.txt—a FASTA file containing all the sequences in which the program could not find one or both MID tags, or the MID combination did not match the table in the input file.
2. FailedInFindPrimers.txt—a FASTA file containing all the sequences in which the program could not find one or both primers, such that only sequences with identifiable MIDs and primers are included in further analysis.
3. FailedInCheckLength.txt—a FASTA file containing all the sequences for which L2 (sequence length between the primers) was not within the allowed range.
4. FailedInQuality.txt—contains all sequences that had identifiable primers at both ends and were within the allowed length range, but the average quality score of the sequence was lower than the input threshold.
5. Log.txt—a tab-delimited text file that assembles details of the run in two parts. The first part (Figure 3A) contains a table of the samples that were sequenced and the following numbers per each sample: total number of sequences found, number of failed sequences in finding primers, percent of the last value out of the total, number of failed sequences in length, percent of the last value out of total, and out of the total after the previous stage, number of sequences with lower quality than threshold, percent of the last value out of total and out of the total after the previous stage, total remaining number of sequences, average quality score. The second part (Figure 3B) contains information regarding the total numbers of the run, such as (partial list): how many sequences failed in the MID tags finding step, how many failed in the primers finding step, how many failed in the length check, how many failed in the quality check, how many are in the sense or antisense orientation, and a short review of the parameters of the run.
6. A text file for each sample, containing the sequences that passed all the checks and were identified as belonging to that sample according to their MID tag combination (given as input, Figure 2).
7. A text file for each sample, containing the quality score sequence of each sequence belonging to the sample, with the scores referring to the gene sequence only (without scores for the MID tags, primers, etc.).

### Ig-HTS-cleaner algorithm

**Figure 4:**
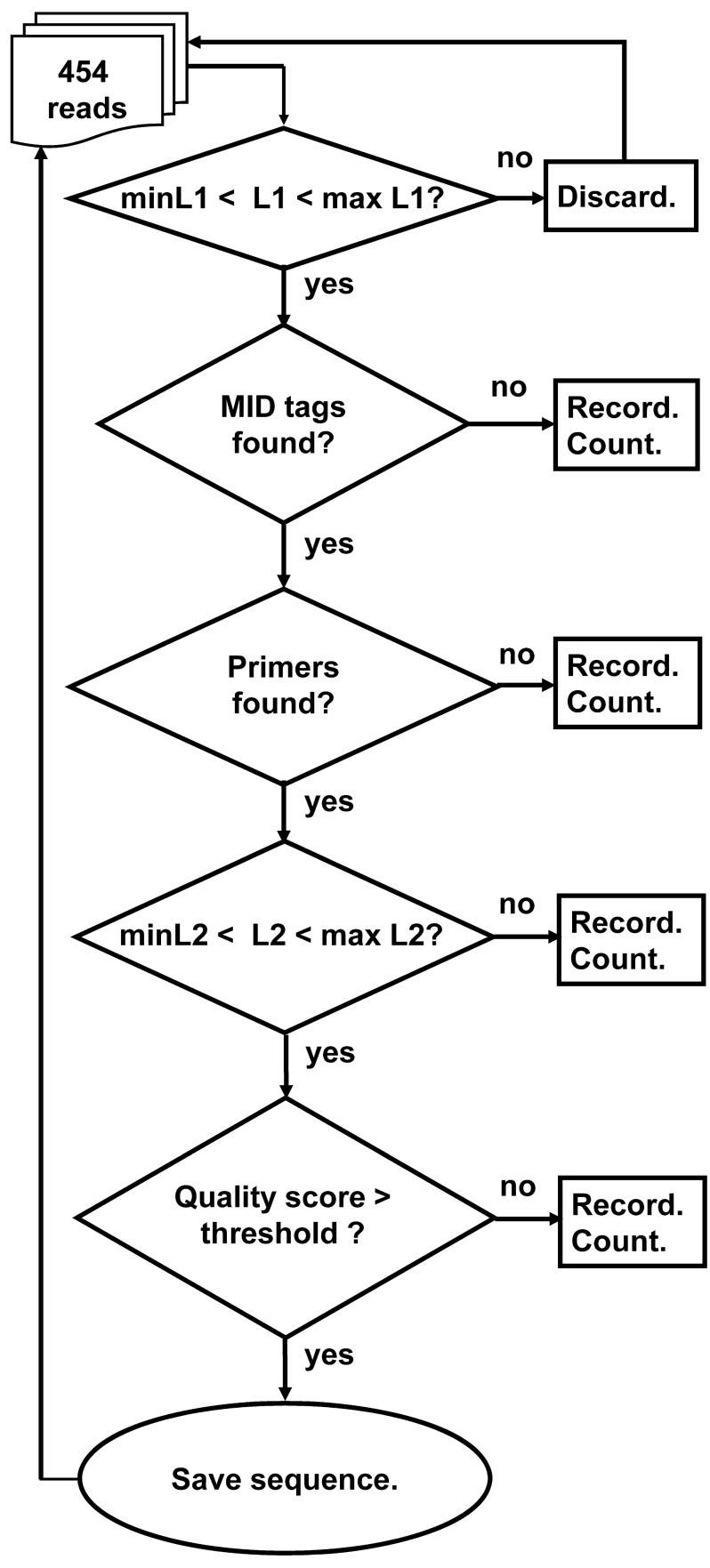
**A schematic outline of the Ig-HTS-Cleaner program algorithm.** “Discard” means: discard the read because it is not likely to be an immunoglobulin gene sequence. “Record” means: write the sequence to a file containing all sequences that failed in this stage. “Count” means: count the number of failed sequences. In case the failure is in the last two stages, the counts are done per sample. “MID tags found?” means: “Is there an identifiable MID tag at each end of the sequence?”, “Primers found?” means: “Is there an identifiable primer at each end of the sequence?” Checks of L1 and L2 are as described in Figures 1 and 2. “Quality score > threshold?” means: “Is the average quality score of the sequence larger than the threshold score given in the input file?” “Save sequence” means: “Write the sequence to the corresponding sample file. Advance the counter of sequences per sample.”

The program works according to the following outline (Figure 4):

1. Read input file and initiate parameters for the run.
2. Read .fna and .qual files and parse them for the run.
3. For each sequence, if it is longer or shorter than the allowed range given in the input file, discard it from analysis.
4. For each sequence that passed the previous step, do the following:
  4.1. Find tags (MIDs) at both ends of the sequence. If found, go to 4.2, else go to 4.5.
  4.2. Find Primers at both ends of the sequence. If found, go to 4.3, else go to 4.5.
  4.3. Check whether the length of the sequence between the primers is within the input range. If so, go to 4.4, else go to 4.5.
  4.4. Check the average quality score of the sequence. If the average quality score is below the input threshold, go to 4.5, else go to 4.6.
  4.5. A sequence that failed in one of the above stages will be written to a discard file according to the reason of failure.
  4.6. A sequence that succeeded in all the above stages will be written to an output file according to its MID combination.

#### MID tag finding

The goal of the first step is to find the MID tags for each sequence. Each 454 run enables the sequencing of several, pooled samples, as long as we mark each sequence according to its sample using known combinations of MID tags at both ends of the sequence (because sequencing may start at each end of the sequence). To use this feature, the genes must undergo preliminary PCR with primers that are connected to specific 10-base oligonucleotides, representing the MID tags. These tags were composed by the sequencing company to provide different oligosequences that are distinguishable. In addition, the reads also contain adapter sequences at their ends that are required for starting the sequencing (Figure 1). Since the number of tags that are sufficiently distinguishable from each other (see below) is limited, one can use primers with a known combination of forward and reverse MID tags for each sample. This way, one can obtain different sequences from many different samples at one run, and later correctly attribute the sequences to the original samples by identifying the MID tag combination in each sequence. If either MID tag cannot be identified, the program would not be able to attribute the sequence to a sample and thus will reject it as a failed sequence. The program searches each sequence for a perfect match to each MID tag (taken from the input file) at both ends of the sequence, which are the regions where we expect the MID tags to be found (and not in the middle of the read, for example). The search is executed on a limited range of nucleotides, given in the input file. If a perfect match is achieved, the number of the tag is noted. If not, the program searches for a perfect match of the minimal MID length that the user has inserted in the input file. This minimal MID length represents the number of consecutive nucleotides of the tag on the side closer to the primers (the inner side of the sequence) that enables unambiguous identification of the tag. This is done because the sequencer more often inserts errors close to the sequence boundaries, so the tags might have been trimmed or contain errors at their outer edges. We found that five consecutive nucleotides are the lowest number of nucleotides that can still distinguish between the different MID tags we used (basic set—Hamming distance: 6, Table 1). However, the minimal MID length is dependent on the Hamming distance of the MID set used, and should be assigned correctly by the user. The program prioritizes a perfect match, thus in case of a perfect match to the minimal MID length of one MID tag, the program will continue the search for a perfect match of the rest of the MID tags. Although rare, in case a perfect match of one MID tag and a perfect match of the minimal MID length of other MID tags are found in the same read-end, the program prefers the perfect match of the full MID tag.

**Table 1:** **List of MID tags, forward and reverse primers used for Ig-HTS-Cleaner validation**^**a**^.

	MID tags (5′ to 3′)^b^	ACGAGTGCGT
		ACGCTCGACA
		AGACGCACTC
		AGCACTGTAG
		ATCAGACACG
		ATATCGCGAG
		CGTGTCTCTA
		CTCGCGTGTC
		TAGTATCAGC
Human	Forward primers (5′ to 3′)	TGCGMCAGGCCCCYGGACAAR
		ARGRAAGGCCCTGGAGTGG
		CCGCCAGGCTCCAGGSAAG
		MGGGAAGGGRCTGGAGTGG
		GAAAGGCCTGGAGTGGATGGG
		TTGAGTGGCTGGGRAGGAC
	Reverse primer (5′ to 3′)	TGACCRKGGTHCCYTGGCCC
Mouse—heavy chain	Forward primers (5′ to 3′)	AGRTYCARCTGCARCAGYC
		TGCAGCTKMAGSAGTCAG
		GARGTGAAGCTKSTSGAGTC
		GAGGAGTCTGGAGGAGGCTT
		CTGGGATATTGCAGCCCTCC
		AGGTGTGCATTGTGAGGTGC
		GTSAGGTGCAGCTKGTRGA
		CAATCCCAGGTTCACCTACAA
	Reverse primer (5′ to 3′)	GTGGTBCCTTSGCCCCAG
Mouse—light chain	Forward primers (5′ to 3′)	MTGATGACCCARTCTCCA
		SRGATATTGTGATGACGCAGG
		AWTGTDCTSACCCARTCTCC
		CCTGTGGRGACATTGTGAT
		AYCCVGATGACYCAGTCT
		CCAGATGTGAYRTYCARATG
		BCAGTGTGACATCCRVAT
		ACACAGGCTCCAGCTTCTCT
		TCCCAGGCTGTTGTGACTC
		CAACTTGTGCTCACTCAGTC
		CTCTAGGAAGCACAGTCAAAC
	Reverse primer (5′ to 3′)	GTGGTBCCTTSGCCCCAG

a Key to degenerate nucleotides: R = A+G; M = A+C; W = A+T; K = G+T; S = G+C; Y = C+T; H = A+T+C; B = G+T+C; D = G+A+T; N = A+C+G+T; V = G+A+C.

b We used the basic set, with Hamming distance = 6.

A legitimate sequence contains a legitimate combination of tags (according to the input file) with the tag at the 5′ end found in the sense orientation and at the one 3′ end found in the antisense orientation. Each search for tags is performed using both the tags and their complementary sequences, in order to identify tags at both ends. If a match of two tags, one at each edge of the sequence, is found, the tag numbers are noted and the sequence in between the MID tags is passed on to the next stage of cleaning. Otherwise, the sequence is discarded from further analysis, and written to a file containing all the sequences that failed in this stage. It is important to note that Ig-HTS-Cleaner can also work in case no MIDs are listed (for example, when the sequencing was carried out on a single sample). The program will then search directly for primers, but it is important that the input file is written properly as detailed in Figure 2.

#### Primer identification

In this stage, the sequence between the MID tags (after MID tags have been removed) is searched for primers at both ends. Again, the search is executed on a limited range of nucleotides, given in the input file. The program runs using the primer lists given in the input file and searches for a perfect match of both forward and reverse primers in the current sequence. The program searches both the primer and its complementary sequence. If one or both primers were not found with a perfect match, the program searches for a partial match between the sequence and the primers, after the latter are trimmed (from the side furthest from the gene) leaving a fraction of the original primers' length, given in the input file (e.g., 75% of the original length). Sometimes PCR and/or sequencing trim the primer ends. Searching only the primer fraction closer to the gene enables the program to identify even primers which contain errors or were trimmed at the ends distal to the gene. This is useful when the reads are from one sample and thus there was no use in MID tags. The partial match allows the number of mismatches per primer defined by the user input. The larger the number of allowed mismatches, the more erroneous primer identifications would occur. Therefore, one should decide on the maximal number of mismatches allowed for the data, according to this trade-off. We examined this parameter on data sets from human and mouse tissues (data not shown). We ran each data set in Ig-HTS-Cleaner with different values between 1 and 10 for the number of allowed mismatches. For our human data set, we found that a value of two allowed mismatches is the best cut-off which minimizes both the loss of sequences and the gain of erroneous sequences with falsely identified primers. For our mouse data set, we found that a value of four allowed mismatches is the best cut-off. This type of analysis may be performed on other data sets with Ig-HTS-Cleaner, to decide on the best value for each data set.

The partial match uses dynamic programming of local alignment of the sequence with each forward or reverse primer, based on the Smith–Waterman algorithm. If both forward and reverse primers are found, the gene orientation is known, hence the MID tag order is known as well, so the sample identity is known. The sequence then proceeds to the next stage. If one or both primers were not found, the sequence is discarded from further analysis, and written to a file containing all the sequences that failed in this stage.

#### Length check

This is the last check before the sequence is accepted as legitimate. If the sequence length between the primers is within the allowed range, the sequence is written to the file of the sample corresponding to its MID tag combination; each file represents one sample. Otherwise, the sequence is discarded, and written to a file containing all the sequences that failed in this stage.

#### Quality check

For each sequence, a file containing the sequencing quality scores per each base is generated during the sequencing run. When using the 454 platform, each nucleotide in each sequence gets a score between 0 and 40 that represents the confidence level of the sequencer that a specific nucleotide is the correct one. In other words, the higher the score per base (or average score per sequence), the better the quality of the sequencing. For each read, a sequence of numbers between 0 and 40 in the original length of the read is generated. The file with all the score sequences is the .qual file.

In addition to cleaning the sequence of tags and primers, a calculation of the quality score per sequence is performed in Ig-HTS-Cleaner using the .qual files. One can either look at each position to investigate its quality (for example, when examining point mutations), or can look at the average quality score per sequence or even per sample to evaluate the quality of sample sequencing (for example, when dealing with preserved tissue samples where DNA is often denaturated). For each sequence, the quality score is calculated as the average score for all bases of the sequence, after tags and primers were removed. Then, the quality score of each sample is calculated, as the average score of the sample's sequences.

**Figure 5:**
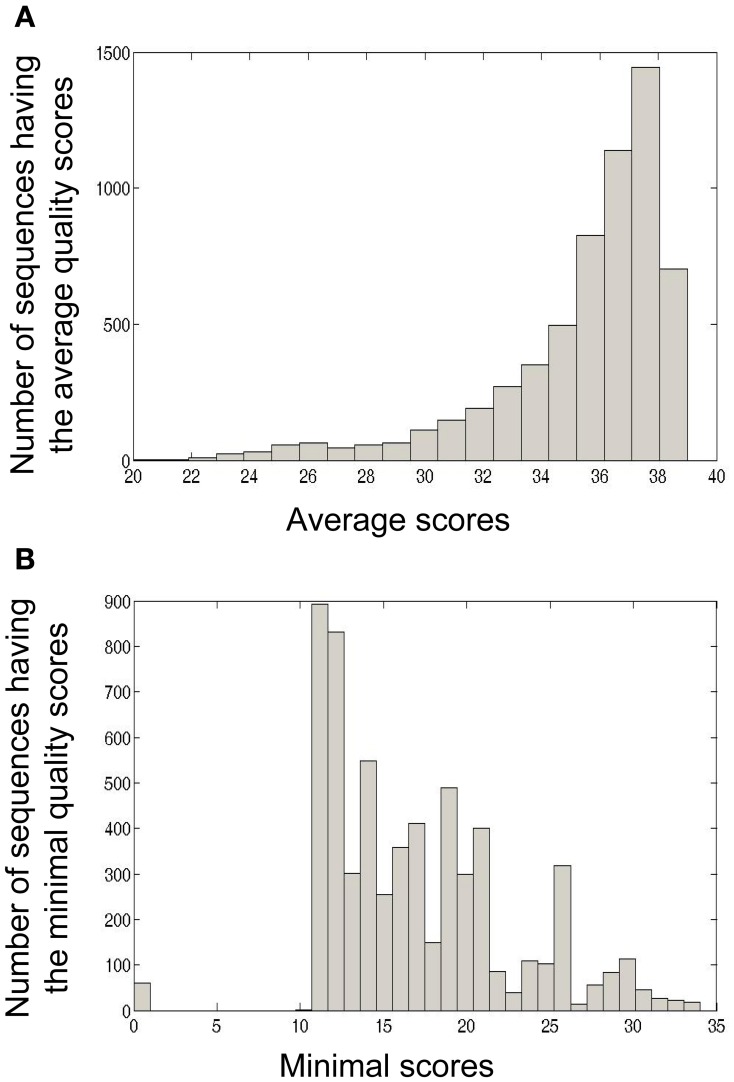
**Four formalin-fixed paraffin-embedded (FFPE) reactive lymph node tissues, archived about 10 years ago, from adult patients, were used as part of a study that dealt with Ig repertoire analysis of gastric lymphomas.** In the current paper we used these samples in order to demonstrate quality scores obtained by the 454 platform using FFPE tissues. **(A)** Average quality score distribution of sequences obtained from four LN samples. For each sequence, the average quality score was calculated as the sum of all base scores divided by the length of the sequence. **(B)** Minimal quality score distribution of sequences obtained from four LN samples. For each sequence, the minimal quality score was calculated as the minimal base score value of all base scores in the sequence.

The calculation of minimal and average quality scores of one sample or a group of samples is also important for downstream analyses. For example, in Ig-Indel-Identifier (see below for more details), point mutations are checked for their quality score, and if the latter is lower than the threshold given by the user, the sequence can be discarded. In Figure 5, we show as an example the quality score analyses of four samples from human lymph nodes (LN).

In this example, many (42.81%) of the sequences in the LN samples had an average quality score of 37–38, which is considered a very high score (Figure 5A). On the other hand, many of the sequences also contained nucleotides with low quality scores (11–12, Figure 5B). These analyses led us to conclude that in our LN data, we could use the average quality score threshold of 30 without losing to many sequences. However, as most of the sequences appear to have a low minimal quality score (mostly lower than 20, with a peak in 11–12), we could not use a minimal threshold larger than 10.

### Ig-indel-identifier input and output

To identify indels created by the sequencer, the sequences are usually compared to some reference gene. In the case of Ig genes, where no reference gene can be used, the sequences should be organized by clones, according to their germline (GL) segment identifications. Then, a consensus sequence can be created for each clone based on all sequences in the clone, and serve as the reference gene. In our analysis pipeline, we first identify the GL segments for every sequence using SoDA (Volpe et al., 2006). Then we group the sequences (from the same sample) that use the same GL segments into one clone, and find the consensus sequence for the N-regions of this clone. In each position of the N-regions, the consensus GL contained the most frequent nucleotide in all the aligned sequences that belonged to the same clone. In the data presented below, we identified clones only based on their V(D)J GL segments for the purpose of demonstration of the action of Ig-Indel-Identifier. However, for proper data analysis, these groups of sequences are aligned and examined, as more than one clone may have the same V(D)J combination. For this purpose, one may use the clustering-based program created by Gaeta et al. (Chen et al., 2010), which deals with groups of sequences that share the same V(D)J segments, and checks whether they belong to one or more clones. After the clonally-related groups of sequences are identified, the sequences from each clone are aligned, along with the “root” sequence composed of the GL segments and N-region consensus, using ClustalW2 (Larkin et al., 2007). The output files from ClustalW2 (^*^.txts files in the PIR format, which is an alignment format) serve as the input files for Ig-Indel-Identifier.

In addition to cleaning artifact indels, Ig-Indel-Identifier identifies point mutations (mismatches) by comparing to the reference gene or GL, and decides whether they are sequencing artifacts or may be derived from natural mutation processes. For each point mutation, Ig-Indel-Identifier checks the quality score of the base, given in the ^*^.qual files. One of the parameters that are given by the user is the minimal quality score. This parameter is used when Ig-Indel-Identifier compares the quality score of the point mutation to the given minimal quality score. If the quality score of the point mutation is lower than the user's threshold, and if this point mutation appears in fewer sequences in the clone than the number given in the input.txt file, the sequence is discarded. Moreover, Ig-Indel-Identifier identifies whether a point mutation occurred inside activation-induced cytidine deaminase (AID) motifs that contain HPT [AACA or the complementary TGTT (MacCarthy et al., 2009)]. If so, the sequence shall not be discarded even if its quality score is lower than the threshold. If the user is not interested in identifying such mismatches, the value of the minimal quality score given in the input.txt file should be set to −1.

Ig-Indel-Identifier receives as input ^*^.txts files, each containing an alignment of a group of clonally-related sequences with their consensus GL sequence; an ^*^.input file, containing all the sequences from the current sample in the FASTA format; and a ^*^.qual file, corresponding to the ^*^.input file, which contains the quality score sequences of the sequences in ^*^.input file. Ig-HTS-Cleaner generates these files automatically. In addition, the user should prepare a file called “input.txt,” with integer values for three parameters, as follows:

1. The minimum number of sequences in a clone that must share the same indel or a low quality score point mutation for this indel or mutation to be considered legitimate. Before discarding a sequence, the user may decide that in case more sequences contain the same indel or low quality score point mutation, the suspected sequence shall not be discarded. The user can choose how many sequences in a clone must share that particular indel or mutation in order to save this sequence. The higher this threshold number, the fewer suspected sequences would be saved.
2. HPT length: the user can decide on the minimum length of same-nucleotide stretch that will be considered a HPT. The longer the HPT length, the more sequences would be saved, since fewer indels would be identified as near-HPT indels.
3. The minimal quality score for a point mutation to be considered legitimate. The higher the minimal quality score, the more sequences would be discarded. If the user is not interested in identifying such mismatches, the value of the minimal quality score given in the input.txt file should be set to −1.

To use Ig-Indel-Identifier on non-Ig gene sequences, one should create a consensus sequence of all sequences that should be checked. This can be done using several programs, such as ClustalW2 (Larkin et al., 2007). A consensus sequence is composed of the most frequent base in each position of the aligned sequences. Then, the consensus and the sequences should be run in ClustalW2 to create the ^*^.txts file that contains the alignment. Ig-Indel-Identifier will work on this file together with the ^*^.input file that should contain the sequences. When using ClustalW2 in order to align each of the sequences with its reference gene, each gap (of one or more nucleotides), in either the GL or the tested sequence, anywhere in the sequence, is designated as an indel that would be checked by Ig-Indel-Identifier.

As output, the program generates the following files for each input file:

1. “(Name-of-input-file)-WithoutIndels.txt”—a FASTA file that contains a list of all sequences from the current input file which contain neither artifact nor uncertain indels.
2. “(Name-of-input-file)-CloneOfSize1WithIndels.txt”—a FAS-TA file that contains a list of sequences that contain suspected indels, but do not belong to a larger clone, so we cannot decide whether each indel is an artifact or not. We call these “uncertain indels,” and keep these sequences separately, so one can perform the analysis either with or without them, as desired.
3. “(Name-of-input-file)-IllegitimateIndels.txt”—a FASTA file that contains a list of the sequences with artifact indels, which are part of a large clone, hence these indels are certainly artifact and not uncertain indels (this list has no overlap with output file number 2).
4. “(Name-of-input-file)-SeqsWithLowQualPointMuts.txt”—a FASTA file that contains a list of sequences that contain point mutations with quality scores lower than the minimal quality score given by the user, and which appeared in fewer sequences in the clone than set by the user.
5. “(Name-of-input-file)-Ig-Indel-Identifier.log”—a file containing the report of the run.

### Ig-indel-identifier algorithm

**Figure 6:**
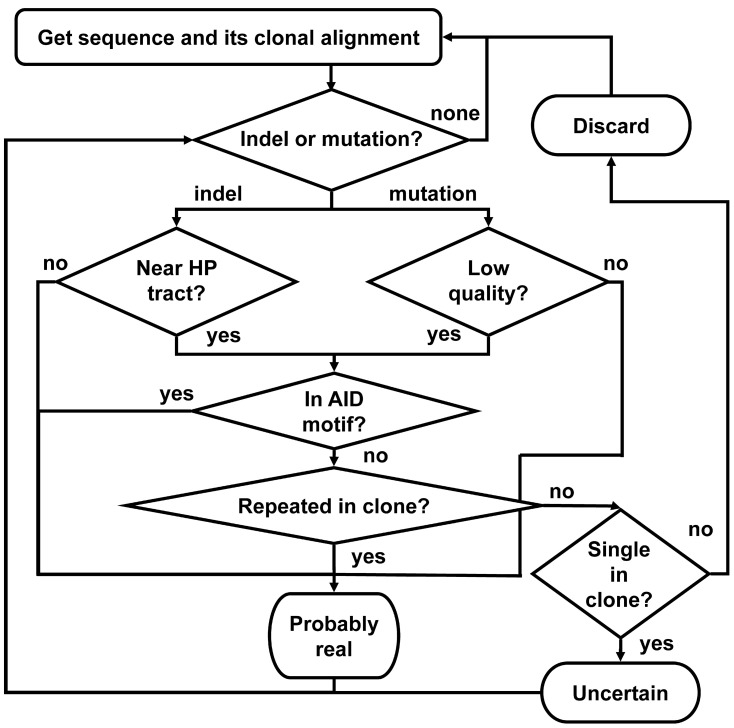
**A schematic outline of the Ig-Indel-Identifier program.** “Indel or mutation?” means: “Are there (more) indels or mutations in the sequence?” “Low quality?” means: “Did the point mutation have a quality score lower than the input threshold?” “Near HP tract?” means: “Is the indel near a HPT?” “In AID motif?” means: “Is the indel or point mutation detected inside the known AID motif (AACA or the complementary TGTT)?” “Repeated in clone?” means: “Are there more sequences in the clone with the same indel?” “Single in clone?” means: “Is this the only sequence in the clone?” “Discard” means: “Discard the sequence because it has an artifact indel.” “Uncertain” means: save the sequence separately as it is suspected to have artifact indel(s), but there are no other sequences in this clone, hence we cannot be sure. “Probably real” means: save the sequence as it has a legitimate indel according to our definitions.

The program works according to the following outline (Figure 6):

1. For each sequence in each clone, do:
  1.1. Search for indels or point mutations by comparing the sequence to the corresponding GL (or consensus) sequence position by position, from the last indel or point mutation checked (or, in a new sequence—from the first position of the alignment) until the alignment ends. If an indel is found, go to 1.2. If a point mutation is found, go to 1.9, else go to 1.7.
  1.2. If this indel appears near a HPT (see below), go to 1.3, else go to 1.6.
  1.3. This indel is suspected to be an illegitimate (artifact) indel. If this indel is unique, i.e., no other sequence in its clone shares the same indel, go to 1.4, else go to 1.6.
  1.4. If the sequence is the single one in its clone go to 1.8, else go to 1.5.
  1.5. The sequence has an artifact indel and hence is discarded from further analyses. Write it to the appropriate file and go to the next sequence (step 1).
  1.6. This indel is considered as a legitimate indel. Go to 1.1.
  1.7. Sequence is OK. Write it to the appropriate file and go to the next sequence (step 1).
  1.8. Sequence has an uncertain indel. Mark as uncertain. Go to 1.1.
  1.9. Check if the quality score of the point mutation base is lower than the threshold given by the user as input. If so, go to 1.10, else go to 1.1.
  1.10. Check if the point mutation is inside the AID motif (AACA or the complementary TGTT). If so, go to 1.1, else go to 1.11.
  1.11. Check the number of sequences in the clone that share the same point mutation. If there are more sequences in the clone but no other sequence shares the same point mutation, go to 1.12. Else, go to 1.1.
  1.12. The sequence has an artifact point mutation and hence is discarded from further analyses. Write it to the appropriate file and go to the next sequence (step 1).
2. Write the sequences to the output files.

In order to decide whether an indel is near or inside a HPT of a length that equals or exceeds the minimum length given by the user, we test the GL sequence to find whether one or more of the following conditions are fulfilled:

- The indel is inside a HPT.
- The indel is 5′ to a HPT.
- The indel is 3′ to a HPT.

We chose to first define HPTs by at least two identical nucleotides. Although this definition is very broad, as pairs of identical nucleotides are very common in all sequences, we preferred to check more indels than to leave sequences with illegitimate ones in the dataset. However, the user can choose the minimal length defining a HPT. If any of the above three conditions is fulfilled, this indel is suspected to be a sequencing artifact. A suspected indel is denoted as an artifact if fewer than the threshold number of sequences that share this indel exist in the clone.

#### Creating a simulated dataset for testing Ig-indel-identifier

In order to check the Ig-Indel-Identifier program, we collected 504 real sequences without indels from previous 454 HTS studies. This dataset contained either groups of clonally-related sequences or single sequences, all already aligned to their GL. We simulated the artificial induction of deletions (see below) on this dataset, creating a new and larger dataset of sequences, each with one deletion at most, near or inside HPTs of different lengths. The simulation was not created in order to reflect the “natural” generation of indels by the sequencer, but simply in order to have sequences with no more than one deletion, near or inside HPTs of different lengths, in order to test the Ig-Indel-Identifier program and analyze the results more easily. The simulation works as follows. For each sequence, the simulation decides whether to introduce a deletion or not. In case a deletion should be introduced, the simulation draws a value (2–10) for the length of HPT that the deletion should occur in. If the sequence does not contain any HPTs with that length, the simulation keeps drawing a value until at least one HPT with the drawn length is found in the sequence. From the list of positions contained in the chosen HPT, the simulation draws one position in one HPT to introduce the deletion in. A deletion (and indels in general) can occur 5′ to the HPT, 3′ to the HPT, or in its middle. Hence, the simulation draws the exact position for the deletion, according to the length of HPT drawn in the first step. Deletions were not allowed at the beginning or at the end of a sequence. Deletions were introduced by replacing the character (A/C/G/T) in the desired position of the aligned sequence by “−”. Ig-Indel-Identifier identifies insertions and deletions according to the alignment of the clonally-related sequences and their GL, and hence a “−” sign in the GL is considered as an insertion, and in the sequence, it is considered as a deletion). Introducing only deletions during the simulation should not affect identification of indels by Ig-Indel-Identifier, and we used only deletions for convenience. After introducing a deletion to the sequence, the simulation draws a value (0–10) for the number of duplicate sequences carrying the same deletion that will be generated. One of our assumptions for correctly identifying artifact indels is that indels that appear in more than one sequence in the same clone are probably real and thus are not designated as suspect. The exact number of sequences carrying the same indel, which are needed for accurately identifying an indel, can change between datasets and observations. It is important to note that all the deletions introduced into the sequences during the simulation were artifact indels, and hence were all expected to be identified by Ig-Indel-Identifier and evaluated based on their frequency within the clone.

The simulation algorithm is as follows:

1. For each sequence (not GL) in a clone do:
  1.1. Decide whether the sequence will undergo a deletion. If so, go to 1.2. Else go to the next sequence (step 1).
  1.2. Draw a value (2–10) for the length of HPTs to search for in the sequence. If no HPT in the drawn value is found, repeat step 1.2. Else, go to 1.3.
  1.3. Draw one HPT from the sequence (there can be several, but only one is chosen).
  1.4. Draw the specific position near or inside the HPT (this depends on the HPT's length).
  1.5. Replace the character in the chosen position (A/C/G/T) with a “−”.
  1.6. Draw the number of duplicate sequences carrying the same indel (0–10).
  1.7. Generate the new sequence(s) and write them into a new file.

## Results

### Ig-HTS-cleaner—performance and validation

We tested the performance of Ig-HTS-Cleaner on real data from 454 HTS. Twenty-nine DNA samples (from a study that will be published elsewhere) were subject to Ig gene amplification by PCR and the products were sequenced on the Roche 454 FLX Titanium platform to yield a total of 44,617 reads. We ran Ig-HTS-Cleaner on this data set with the following parameters: average quality score threshold of 20, 2 allowed mismatches in the primer search, 75% of the primer's length to search, and a range of 50 bases at the ends of the read for the MIDs and primers search (denoted as “combination 1,” see Table 2). Out of the 44,617 reads, 35,453 reads contained MID tags at both ends of the read. In the next step, Ig-HTS-Cleaner discarded 2504 sequences that did not contain identifiable primers at both ends, because in such sequences we cannot identify sequence orientation. It is important to identify primers at both ends, not only in order to identify where the gene is positioned inside the read, but also to identify the orientation of both primers, in order to discard those chimeric sequences—created during the PCR or the sequencing—that contain both primers in the same orientation rather than opposite orientations. These artifact sequences can be identified by primers with the same orientation. Only one read did not have a length within the requested range and was discarded. The reason the latter number was so low is that all Ig genes are of similar lengths, such that if the whole gene between the primers was sequenced, it is highly likely to have the correct length. Much shorter or longer reads could be chimeric sequences (discussed below). Only seven sequences did not pass the average quality threshold, which we set to be 20. This is not surprising, as most of the nucleotides were sequenced with a quality score of 20–40 (see Figure 5). The user may decide whether to assign this threshold a higher value, and thus to discard more sequences. Finally, when Ig-HTS-Cleaner had finished running, we were left with 32,941 remaining sequences (Table 2, parameter combination 1). The list of MID tags and primers used in this specific run can be found in Table 1.

**Table 2:** **A summary of the Ig-HTS-Cleaner results in each parameter combination: human data set of 44K sequences**.

**Parameter combination**	**Number of sequences received**	**Number of sequences with tags**	**Number of sequences without primers**	**Number of sequences that failed due to length**	**Number of sequences that failed in quality check**	**Number of remaining sequences**
1	44,617	35,453	2504	1	7	32,941
2	44,617	35,891	1662	3	11	34,215
3	44,617	35,114	3528	1	3	31,582
4	44,617	36,246	439	0	8	35,799
5	44,617	35,911	1684	0	4	34,223

See text for parameter combinations.

We ran Ig-HTS-Cleaner on the same data set with four different combinations of parameters (numbered 2–5 in Table 2), in order to demonstrate the influence of each parameter on the cleaning process. Parameter combination number 2 had the same parameter values as the original run (number 1) except for allowing up to 4 mismatches. It is not surprising that fewer reads were discarded in the stage of primer search (because more mismatches were allowed), thus there were more sequences attributed to MID tag combinations. In addition, six reads were included due to the lenient primer search, but two were discarded due to insufficient length, and 4 were discarded due to a low average quality score. Parameter combination number 3 had the same parameter values as the original run (number 1) except for the fraction of primer to search, which was set to 100%—that is, the program would search only for the full primer. It was obvious that in this case, more reads would be discarded, as we searched for the full primer sequence and allowed only 2 mismatches. There were fewer reads with low average quality scores, because some were already discarded in the primer search step. Parameter combination number 4 had the same parameter values as the original run (number 1) except for the range of 25 bases at the ends of the read for the MID and primers search. In this case, which took longer than previous runs (see below regarding run times), more reads were subjected to partial match, which allows mismatches, and thus fewer reads were discarded. Parameter combination number 5 had the same parameter values as the combination number 4 except for the fraction of primer to search, which was set to 100%. In this case, more reads were discarded than in the previous run, because the program searched only for the full primer. However, fewer reads were discarded than in runs 1 and 3, because more reads were subjected to partial match.

Validation of Ig-HTS-Cleaner results was done manually. Each cleaning step was examined individually. We checked for false negatives by looking at 50 reads that were discarded due to lack of MID tags and manually checked whether they do contain MID tags. None of the sequences was found to be false negative in this step. False positives were also not found when we performed manual checks on about 10 sequences from each sample (a total of ~200 sequences), which had successfully passed this step. The same validation steps were performed on ~150 sequences lacking primers and on ~100 sequences at both ends of which primers were found. This step was more complicated due to the use of dynamic programming for identifying primers with less than 100% match. About 50 sequences that did not contain 100% match of a primer were also checked to validate the dynamic programming algorithm's accuracy. All of the sequences that proceeded to the next cleaning step contained primers at both ends. Discarded sequences in this step indeed lacked one or more primers at their edges. Length checks were done automatically: a simple script validated that the lengths of all sequences that had passed the length check are truly within the allowed range, and that the sequences that had failed this step really were outside the allowed length range. We also validated that the sequences that were discarded due to lower quality score had indeed an average quality scores below the threshold. We collected these sequences and automatically calculated their average quality scores.

Applying Ig-HTS-Cleaner on the first data set of 44,617 reads, with a range of 50 nucleotides at each end to search primers in, took approximately 3–4 min to run on an Intel® core™2 CPU 6700, 2 GB RAM 2.67 GHz. When the primer search range was decreased to 25, Ig-HTS-Cleaner run took almost 1 h on the same computer. The longer run time is because when the primer search range decreases, primers that were located not in the first 25 bases but closer to the inner side of the sequence would not be found. Hence, the program would search for partial match of the primer(s), and that would take much longer. We then proceeded to test Ig-HTS-Cleaner on larger data sets, obtained from human and mouse DNA samples and together representing ~527,000 reads that were assigned into samples. However, for this number of reads we could not use the above-described computer due to memory shortage, and needed to run Ig-HTS-Cleaner on our UNIX server, which is equipped with larger RAM (16 GB). An Ig-HTS-Cleaner run on the ~527,000 reads took approximately 5 min on our UNIX server. Hence, we recommend using Ig-HTS-Cleaner on UNIX machines or on PCs with large internal memory. Regarding memory complexity, the program saves the reads for the whole running time in a special data structure, representing O(*n* × *k*) memory, where *n* represents the length of a sequence, *k* represents the number of sequences, and *n* « *k*, hence O(*k*) memory is required. For each instance of dynamic programming used in finding a partial primer match, we have O(*n* × *m*), where *n* represents the sequence length and *m* « *n* represents the primer length. Actually, when the program searches for the primer, it searches it in a window shorter from the full sequence length at each side of the sequence, and not in the whole sequence, as we expect the primers to be on the sides of the sequence and not in the middle. Thus, O(*n* × *m*) is limited to a finite number. Moreover, a partial match search was carried out in less than 10% of the reads, reducing the complexity in one order of magnitude. To summarize, a run of ~500,000 sequences performed on a computer equipped with large internal memory (16 GB) would yield results within a short time (5 min). Table 3 presents the cleaning results of both human and mouse data sets with an average quality score threshold of 20, and 2 and 4 allowed mismatches of primers for the human and mouse data sets, respectively. The two data sets were sequenced in the same run, but in different lanes. We present here the numbers of sequences attributed to each dataset and the cleaning results using Ig-HTS-Cleaner. The list of MID tags and primers used in this specific run can be found in Table 1.

**Table 3:** **A summary of the Ig-HTS-Cleaner results: human and mouse data sets of 500 K sequences together**.

**Organism**	**Number of sequences with tags**	**Number of sequences without primers**	**Number of sequences that failed in due to length**	**Number of sequences that failed in quality check**	**Number of remaining sequences**
Human	116,546	3248	4	10	113,284
Mouse	410,352	143,729	271	4	266,348

### Ig-indel-identifier—performance and validation

We tested the performance of Ig-Indel-Identifier on the first dataset described above. The original study included 29 samples, but after cleaning with Ig-HTS-Cleaner, five samples out of the 29 yielded fewer than 30 sequences each, and these sequences were discarded from further analyses due to lack of interest. Data from 24 samples, which originally contained 36,944 sequences, were taken from the output of Ig-HTS-Cleaner. Out of these sequences, 33,767 sequences did not contain indels at all; this is reasonable, since SHM inserts mostly single base substitutions (Liu and Schatz, 2009; Steele, 2009). On the other hand, 3177 sequences contained indels (both uncertain and artifact), representing 8.6% of all sequences. Of the latter, 93 sequences with uncertain indels and 3084 with artifact indels were found (Table 4).

**Table 4:** **Numbers of sequences after Ig-Indel-Identifier cleaning**.

**Total**	**Number of sequences w/o indels**	**Total number of sequences with indels**	**% of sequences with indels**	**Number of uncertain indels**^**a**^	**Number of sequences with artifact indels**^**b**^
36,944	33,767	3177	8.6	93	3084

a An uncertain indel is an indel in a single sequence that does not belong to a multi-sequence clone.

b An artifact indel is an indel near a HPT, where no other sequences in the same clone contain the same indel (and is not in a single sequence).

Applying Ig-Indel-Identifier on the data set of 44,617 reads took approximately 5 min to run on an Intel® core™2 CPU 6700, 2 GB RAM 2.67 GHz. Regarding memory complexity, the program saves the reads for the whole running time in a special data structure, representing O(*n* × *k*) memory, where *n* represents the length of a sequence and *k* represents the number of sequences. For each sequence, both the sequence and its GL (consensus) sequence are being compared, representing additional O(*n*) memory space.

### Testing Ig-indel-identifier on simulated data

We collected 504 sequences without indels, from 85 clones with sizes ranging from a single sequence to 73 sequences. These sequences were taken from seven different samples from the dataset described above. The simulation ran on each clone 10 independent times, each time with different random variables as described above, in order to extend the dataset. These simulations yielded a total of 10,475 sequences, out of which 10,308 sequences had artifact deletions.

We then ran Ig-Indel-Identifier on each clone from the simulated dataset, using all possible combinations of the following program parameters: the minimum HPT length (with values ranging between 2 and 5) and the required number of sequences sharing the same indel for an indel to be considered a legitimate indel (with values ranging between 1 and 12). The former value range was based on our finding that there were no HPTs of length higher than five in our dataset.

For each parameter combination, we recorded how many artifact deletions were identified and calculated the percentage of accuracy (the number of identified deletions divided by the total number of artifact deletions and multiplied by 100).

As expected, the higher the value for HPT length, the lower the number of identified artifact deletions. This is reasonable since the probability of a HPT to be found in a sequence decreases with its length (HPTs of length two are much more frequent than HPTs of length five).

On the contrary, but also as expected, the higher the required number of sequences sharing the same deletion, the more artifact deletions were identified, designated as suspect and finally discarded. This is also reasonable, since the higher the required number of sequences sharing the same indel, the more stringent the requirements, and hence more indels (and sequences) do not fulfill these requirements.

Table 5 presents the average required number of sequences sharing the same deletion in each HPT length that was needed to identify 50% of the artifact deletions, or to identify all the artifact deletions—or as many as the program managed to identify. Due to the fact that Ig-Indel-Identifier was run individually on different samples (in our case we used seven different samples to collect the initial dataset of clones and sequences without indels from), and since each Ig-Indel-Identifier run included all combinations as explained above, the results in Table 5 represent the average numbers out of 336 (7 × 4× 12) runs.

**Table 5:** **The numbers of sequences sharing an indel within a clone that are required in order for it to be considered legitimate, that allow the indicated level of artifact indel identification**.

**HPT length**	**50% identification**	**Maximal identification**
2	5	10
3	7	10
4	–	9^*^
5	–	7^*^

* Numbers marked with a “^*^” indicate that for the indicated HPT length, the program achieved less than 50% identification even with the indicated number of required sequences. Higher values gave the same results, so the minimal values of the required number of sequences in a clone that share the same indel were chosen.

When the minimal HPT length was 2, 50% of the artifact deletions were identified only when we required more than five sequences sharing the same deletion (on average) for a deletion to be considered legitimate. The maximal number of the artifact deletions identified out of the total deletions in the dataset was obtained only when we required more than 10 sequences sharing the same deletion (on average). Similarly, when the HPT length was 3 (or 4), 50% of the artifact deletions were identified when we required more than 7 (or 9) sequences sharing the same deletion (on average). For HPT = 3, the maximal number of the artifact deletions identified out of the total deletions in the dataset was when we required more than 10 sequences sharing the same deletion (on average). With HPTs of length 4–5, no required number of sequences sharing the same indel could help identify more than 50% of the artifact indels. If the HPT length is set to 4 in Ig-Indel-Identifier, the program does not consider HPTs of length less than 4 and hence “misses” those indels, which brings the % identification down no matter now many sequences we require. When the HPT length was 5, the maximal number of the artifact deletions identified out of the total deletions in the dataset was when we required more than seven sequences sharing the same deletion (on average). Again, most of the deletions occurred near or inside HPTs of length less than 5, thus, no matter what the number of sequences sharing the same indel was, most of the indels were missed. Based on these results, our conclusion is that one should consider using either 2 or 3 as the values for HPT length in Ig-Indel-Identifier; otherwise the program would miss many artifact indels. Of course, it would be more efficient for each user to investigate their data for appearances of indels near or inside HPTs and lengths of the latter, in order to decide on the appropriate parameter values. We also recommend demanding as many sequences to share the same indel as possible (each user should optimize this number for their specific dataset).

In addition, we tested Ig-Indel-Identifier performance on 2355 Ig Sanger sequences from data published on B cells from autoimmune diseases (AI) (Zuckerman et al., 2010a,b) and lymphomas (Zuckerman et al., 2010c). The Sanger sequences barely contained indels (Table 6). However, when they did, most of the indels were uncertain, due to the small numbers of sequences sampled using the Sanger method. Only a small proportion of the sequences contained artifact indels. Therefore, the many indels observed in the 454 sequences and identified by Ig-Indel-Identifier are probably sequencing errors.

**Table 6:** **Numbers of Sanger and 454 sequences after Ig-Indel-Identifier cleaning**.

**Sequencing method**	**Sample (number of sequences in the sample)**	**Number of indels found in the sample**	**Number of sequences with artifact indels**^**a**^	**Number of uncertain indels**^**a**^	**Number of sequences w/o indels or with legitimate indels**	**Total number of point mutations in the sample**	**Number of point mutations in AID targeting motifs**
Sanger	MG (33)	24	2	0	31	459	11
	MS (78)	4	0	0	78	2709	25
	Myositis-Bradshaw (33)	25	4	0	29	781	0
	RA-Gause (28)	7	1	2	25	356	6
	RA-Miura (123)	125	10	73	41	2495	29
	SS-Gellrich (70)	2	0	0	70	1269	13
	SS-Jacobi (190)	94	14	58	118	1226	22
	BL-Chapman (22)	0	0	0	22	465	1
	MZL-Zhu (72)	0	0	0	72	587	8
	DLBCL (708)	98	17	0	692	19,416	209
	FL (772)	87	31	0	741	19,249	226
	PCNSL (226)	4	2	0	224	7204	127
454	LN-1 (507)	2276	24	1	482	4631	2
	LN-2 (913)	8164	82	1	837	5682	0
	LN-3 (585)	5803	30	0	557	3019	0
	LN-4 (1137)	17,831	280	2	887	5744	0

a Same as in Table 4.

MG, Myasthenia Gravis; MS, Multiple Sclerosis; RA, Rheumatoid Arthritis; SS, Sjögren's Syndrome; BL, Burkitt's Lymphoma; MZL, Marginal Zone Lymphoma; DLBCL, Diffuse Large B Cell Lymphoma; FL, Follicular Lymphoma; PCNSL, Primary Central Nervous System Lymphoma; LN, Lymph Node.

For the original studies from which the sequences were taken, see Zuckerman et al. (2010a,b,c).

## Discussion

HTS is increasingly popular in various research fields such as immunology, cancer research, and evolutionary biology. The enormous amounts of data generated by HTS require the development of new and efficient data processing algorithms. There are already several tools for cleaning and analysis of HTS data, but no dedicated program for Ig genes has been made publicly available up to this work. In this paper, we present Ig-HTS-Cleaner, a program that successfully performs the pre-processing of Ig sequences derived from HTS, and Ig-Indel-Identifier, a program that precisely distinguishes between legitimate and artifact indels which are typical of 454 HTS (and discards the latter), and also discards sequences containing point mutations with low quality score that appear only once in a clone. The two programs are independent of each other or any other tools, and are applicable to other sequences, in addition to Ig genes, and other sequencing platforms in addition to 454.

While the rules defined in Ig-Indel-Identifier do not guarantee that we identify all sequencing artifacts, it is known that HTS using the 454 platform mostly introduces indels near HPTs (Huse et al., 2007), while SHM of Ig genes mostly introduces point mutations rather than indels (Liu and Schatz, 2009; Steele, 2009). Moreover, most features of the SHM process are studied through analysis of point mutations (Zuckerman et al., 2010a,b,c). Thus, on one hand, the elimination of a large fraction of the artifact indels helps us retain the legitimate sequences that—having fewer indels—are easier to align and analyze further. On the other hand, the remaining artifact indels that may have not been eliminated do not affect the measurements of mutation characteristics.

The next step in analyzing Ig genes is to identify the GL V(D)J segments used in the unmutated (ancestor) sequences, and then assign the sequences into clonally-related groups. For gene segment identification, we use either SoDA (Volpe et al., 2006) or iHMMune-align (Gaëta et al., 2007). Our automated pipeline, which follows the above-described processes of cleaning the data, removing indels and identifying GL segments, contains our program “Ig_Clone_Finder^©^,” which groups the sequences into clones based only on their V, D, and J segments. The weakness of this method is that two different rearrangements using the same V(D)J segments may be grouped together into the same clone; this necessitates manual checking of groups that clearly segregate into two or more different clones. A more sophisticated method, based on sequence clustering and the use of an empirical cut-off, was recently published by Chen et al. (2010); however, it has yet to be tested on large data sets.

Another weak point of HTS analysis is identifying chimeric (hybrid) sequences generated during PCR or HTS. It is essential to discard such sequences before performing repertoire and hypermutation analyses. Although there are a few existing tools that identify and discard chimeric sequences from HTS data (Huber et al., 2004), these programs are not suitable for use with Ig gene sequences. These programs depend on reference sequences that do not exist for Ig gene sequences, due to the complexity of Ig gene rearrangements and mutations, which make almost each sequence unique. There are several reasons why such a tool has not been fully developed yet for Ig genes, although HTS is already available and is extensively used. One major reason is the large homology between V segments. In order to find a chimeric sequence, one must recognize the two most probable V segments [obtained by e.g., SoDA2 (Munshaw and Kepler, 2010) or iHMMune-Align (Gaëta et al., 2007)] that are likely to have been merged to create the suspected sequence. The problem is that GL genes from the same family can only diverge by up to 25% (by definition) and are usually much more similar, while mutated Ig genes can diverge by several tens of mutations (Cook and Tomlinson, 1995; Rajewsky, 1996). Thus, it is often impossible to identify whether the mismatches in the alignment are due to SHM of the suspected sequence, or due to SNPs that distinguish between the two almost identical V segments. Due to these reasons, we still search for chimeric sequences manually. However, discarding sequences that are too short or too long to be legitimate Ig gene sequences, as we do, probably gets rid of some obvious chimeras.

Technologies for HTS and the amounts of sequences generated using them continue to evolve. For this reason, we developed Ig-HTS-Cleaner and Ig-Indel-Identifier, two independent programs for cleaning high-throughput sequences. We hope these programs would be useful to the research community.

### Conflict of interest statement

The authors declare that the research was conducted in the absence of any commercial or financial relationships that could be construed as a potential conflict of interest.
